# The importance of autopsies despite the declining number amidst the COVID-19 pandemic

**DOI:** 10.4322/acr.2021.371

**Published:** 2022-04-20

**Authors:** Monika Bhatt, Mani MovaseghiGargari, Momal T Chand

**Affiliations:** 1 Ascension St. John Hospital and Medical Center, Department of Pathology, Detroit, MI, USA; 2 St. George’s University School of Medicine, St. George’s, West Indies, Grenada

Dear Editor:

Reported for the first time in Wuhan, China in late 2019, coronavirus disease 2019 (COVID-19) has since taken the world by storm.^1^^,^ 2 Caused by severe acute respiratory syndrome coronavirus 2 (SARS-CoV-2), the disease’s rapid worldwide spread led the World Health Organization to declare COVID-19 a global pandemic on March 11, 2020.^1^^,^ 2

As the number of COVID-19 infections has soared throughout the world, no industry has been spared from its effects. The healthcare field has faced a substantial burden from the pandemic. In addition to impacting frontline workers, the pandemic has also put a strain on undergraduate and graduate medical education,^3^ including pathology residency programs. Here, we discuss the effects of the COVID-19 pandemic on decreasing the number of autopsies performed by pathology resident physicians^4^ and argue for the continued importance of autopsy training in pathology residency programs.

An autopsy is the external and internal investigation of the deceased followed by microscopic evaluation by a pathologist of tissue representatives from the internal organs. The purpose of hospital autopsies performed by residents in anatomic pathology residency programs, aside from practicing the skills necessary to perform an autopsy, is to elucidate any information that may provide clinical clues about a patient’s demise that may not have been known prior to the patient’s death.^5^ Although the demand for autopsies has been decreasing for likely a multitude of reasons, among them the advancements in medical technologies that may allow physicians to more confidently make clinical judgments and diagnoses prior to a patient’s death, they were previously the only source of internal investigation and remain the most accurate modality for determining cause of death.^6^

For years, autopsies have consistently played a crucial role in medicine. They have deepened and continue to deepen our understanding of the pathophysiology of innumerable disease processes^4^ that have led to better management of patients, targeted therapies, and clinical outcomes. In respect to anatomic pathology residency, autopsies are a required part of training and are, therefore, important for residents, as they allow pathology trainees the ability to practice the skills needed to be board-certified anatomic pathologists. Moreover, they enrich the educational experience by offering residents the unique opportunity to clinically correlate gross external and internal examination to benign and malignant histopathological findings.

Despite their significant contributions to science and medicine, the importance of performing autopsies in pathology residency training was questioned in the 2014 meeting of the Association of Pathology Chairs as the steadily decreasing autopsy rate was perceived as an indication to replace autopsy training with newer topics like molecular genetics and informatics.^7^ After two years of research by the Autopsy Working Group, it was decided that autopsy training will remain a part of pathology residency training.^7^ Subsequently, the ACGME continued to require that pathology residents in anatomic pathology residency programs perform a minimum of 50 autopsies to qualify to take the American Board of Pathology (ABPath) examination.^8^

Even with the reinstated importance of autopsies, autopsy rates have steadily decreased, creating concern for pathology residencies across the nation from the uncertainty of whether their residents will be able to achieve the number of autopsies mandated by ACGME. While the overall number of annual deaths has increased from 1.9 to 2.2 million in the United States from 1972 to 1994, the rate of autopsies has declined from 19.1 to 9.4%, respectively.^9^ A study over the next nine years showed a continuing downward trend in the national average autopsy rate to 8.3% in 2003.^9^ This decrease is not unique to the United States; a decline in autopsy rates is also seen globally. Reports show autopsy rates in Germany, France, Sweden, and the United States have declined by 30% or more since 2005.^10^

To help programs mitigate concerns about meeting the required autopsy number during pathology residency, ACGME permits two residents to share an autopsy.^8^ While this sanction aids in making the autopsy requirement more attainable for residents, concerns amplified during the COVID-19 pandemic. Because of the uncertainty in the potential risk of exposure during autopsy performance, such as the degree of infectivity produced by SARS-CoV-2 aerosols from lung dissection,^4^ and constrained personal protective equipment (PPE) supply, pathology residency programs nationwide took the stance of declining COVID-19 positive autopsies early in the pandemic. This was especially the case in hospitals that were not equipped with adequate resources and environmental controls, such as proper morgue ventilation, to safely perform autopsies.^11^ Moreover, many hospitals likely did not perform autopsies on patients that expired from COVID-19, since the cause of death often was known to be related to respiratory failure.^4^ Therefore, the pandemic further contributed to the already low autopsy rates adding to the challenge of pathology residents fulfilling the autopsy requirement.

Some relief came in August 2020, when ACGME assessed the current state of affairs and lowered the autopsy number pathology residents are required to complete during their residency training from 50 to 30.^12^ Although the lowered mandated number has provided more ease in meeting the graduation and board criterion, it may signal to the pathology community a depreciating value of autopsy training in pathology residency. However, as discussed by Waidhauser et al.^10^ there remains a high percentage of “major discrepancies between clinical and autopsy diagnosis.” This is very much in contrast to what one may expect. As significant advances are being made in medical technology, one may anticipate there to be a decrease in differences between clinical and autopsy cause of death findings.^10^ Nevertheless, this is still not the case. A study from Augsburg, Germany found that in 67 autopsies with an oncologic history, there were major discrepancies in the cause of death between the clinical and autopsy diagnoses 51% of the time.^10^ As autopsies remain the most accurate and specific method for elucidating exact disease pathology causing death,^6^ they continue to be imperative in the disease workup process in modern medicine. This remains the case, even with technological advancement, including diagnostic imaging in the post-mortem setting.

Consequently, their ability to enhance our understanding of the pathophysiology of diseases and their sustained importance as the gold standard in determining the most accurate and precise cause of death illustrates the continued importance of autopsies in medicine. The value they provide emphasizes why it should continue to remain a requirement for pathology residency and be incorporated in the anatomic pathology board exam. Failure to adequately provide high-quality autopsy training to pathology resident physicians will deprive them of imperative skills required to become well-rounded pathologists and deprive the healthcare system of a key asset in comprehending disease processes and determining cause of death.

## References

[1] Kumar J, Makheja K, Rahul F (2021). Long-term neurological impact of COVID-19. Cureus.

[2] Nawaz M, Rivera E, Vinayak S (2021). Comparison of sexual function before and after COVID-19 infection in female patients. Cureus.

[3] Mabis C, Chand MT, Miller S (2021). Utilizing electronic resources to promote your residency program. Autops Case Rep.

[4] Cao W (2021). Autopsy education and rate: effect of the COVID-19 pandemic. RI Med J.

[5] Shojania KG, Burton EC (2008). The vanishing nonforensic autopsy. N Engl J Med.

[6] Inai K, Noriki S, Kinoshita K (2016). Postmortem CT is more accurate than clinical diagnosis for identifying the immediate cause of death in hospitalized patients: a prospective autopsy-based study. Virchows Arch.

[7] Davis GG, Winters GL, Fyfe BS (2018). Report and recommendations of the Association of Pathology Chairs’ Autopsy Working Group. Acad Pathol.

[8] Hamza A (2017). Declining rate of autopsies: implications for anatomic pathology residents. Autops Case Rep.

[9] Nemetz PN, Tanglos E, Sands LP, Fisher WP, Newman WP, Burton EC (2006). Attitudes toward the autopsy: an 8-state survey. MedGenMed.

[10] Waidhauser J, Martin B, Trepel M, Märkl B (2021). Can low autopsy rates be increased? Yes, we can! Should postmortem examinations in oncology be performed? Yes, we should! A postmortem analysis of oncological cases. Virchows Arch.

[11] Siefring C, Sachire J, Thomas D, Allenby P (2020). Exposure reduction in COVID-19 autopsies. Autops Case Rep.

[12] The American Board of Pathology (ABPath) (2020). New certification requirement for autopsies.

